# Beta‐blockers exert potent anti‐tumor effects in cutaneous and uveal melanoma

**DOI:** 10.1002/cam4.2594

**Published:** 2019-10-07

**Authors:** Prisca Bustamante, Denise Miyamoto, Alicia Goyeneche, Paulina García de Alba Graue, Eva Jin, Thupten Tsering, Ana Beatriz Dias, Miguel N. Burnier, Julia V. Burnier

**Affiliations:** ^1^ Cancer Research Program Research Institute of the McGill University Health Centre Montreal QC Canada

**Keywords:** adjuvant therapy, beta‐blockers, melanoma, uveal melanoma

## Abstract

**Background:**

Melanoma is a life‐threatening group of cancers mainly affecting the skin (cutaneous melanoma, CM) and the eyes (uveal melanoma, UM). Nearly half of patients with UM develop liver metastases regardless of the primary treatment. For this reason, adjuvant therapy to prevent disease progression is essential to improve survival of patients with melanoma. Beta‐adrenoceptors (β‐AR) have emerged as novel targets to inhibit tumor growth and dissemination in CM, but have not been investigated in UM.

**Methods:**

The aim of this study was to comprehensively evaluate the effects of a non‐selective β‐blocker in UM and CM. Propranolol was tested on four UM and two CM cell lines to determine the effects of this beta‐blocker. The expression of β‐AR in UM was assessed in enucleated eyes of 36 patients.

**Results:**

The results showed that propranolol exerts potent anti‐proliferative effects, attenuates migration, reduces VEGF and induces cell cycle arrest and apoptosis in both UM and CM in a dose‐dependent manner. Furthermore, levels of cell‐free DNA released from the cells correlated to propranolol treatment and may be an indicator of treatment response. Finally, immunohistochemical analysis revealed the expression of β1 and β2 adrenoceptors in all UM patients, with higher expression seen in the more aggressive epithelioid versus less aggressive spindle cells.

**Conclusions:**

Collectively our data suggest that a nonselective beta‐blocker may be effective against melanoma. For the first time, we show potent anti‐tumor effects in UM cells following propranolol administration and expression of β1 and β2 adrenoceptors in patient tissue.

## Graphical abstract

For the first time, we show potent anti‐tumor effects in uveal melanoma (UM) cells following propranolol administration and positive expression of β1 and β2 adrenoceptors in all human UM specimens, correlating with aggressiveness of the tumor. Collectively our data suggest that a nonselective beta‐blocker may be effective against melanoma, and further studies are warranted to validate this as an adjuvant therapy in melanoma.

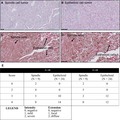

## 1 INTRODUCTION

Ocular melanoma is the most common primary intraocular malignancy in adults and the second most common type of melanoma. It largely arises from melanocytes of the uveal tract (uveal melanoma, UM).^1^ While local control of UM by enucleation or local radiation is effective, approximately 50% of patients will develop metastasis,^2^ primarily to the liver. Patients with metastatic UM have an estimated survival of 6 months.^3^, ^4^ There is a crucial need to better understand the mechanisms involved in tumor dissemination and develop new sustainable and effective adjuvant therapeutic options. Drug repurposing studies are a cost‐effective means to find new applications to approved drugs with good safety profiles.

Beta‐adrenoceptors (β‐AR) have recently emerged as novel targets to inhibit melanoma growth and dissemination. Beta‐adrenoceptors are membrane receptors activated by catecholamines, such as epinephrine and norepinephrine. These stress‐related hormones are increased in patients with cancer and their contribution to tumor growth and disease progression has been established^5^ including in melanoma.^6^ Once activated by catecholamines, β‐AR stimulate several intracellular signal transduction pathways, such as the nitric oxide synthase, related to melanoma development and progression.^7^ Primary downstream effects include vasodilation and release of pro‐angiogenic factors, such as vascular endothelial growth factor (VEGF).^5^

The therapeutic potential of a non‐selective β‐blocker in cutaneous melanoma (CM) progression has been partially evaluated. A retrospective study demonstrated that patients diagnosed with CM who were regularly using β‐blockers had less disease progression and a lower mortality rate than patients not exposed to the drug.^8^ This was confirmed in a recent clinical trial, which assessed the effect of off‐label propranolol in patients with localized CM throughout a 3‐year follow‐up.^9^ Previous studies have demonstrated that all three subtypes of β‐AR are expressed in CM tumoral cells and in its microenvironment.^10^ Stimulation of β‐AR induces cellular proliferation, matrix metalloproteinase synthesis, and release of pro‐angiogenic cytokines.^6^, ^11^ Likewise, in vitro experimental models using human CM cells and in vivo animal models have shown that these parameters are inhibited once β‐ARs are blocked.^12^

Propranolol is a nonselective β1 and β2‐AR blocker that has been in use since 1964 to treat coronary insufficiency.^13^ In addition to its beneficial effects on the cardiovascular system, propranolol has also been successfully used for other purposes, such as glaucoma, migraine prophylaxis, and portal hypertension. Due to its anti‐proliferative properties, propranolol has become the first therapeutic choice for infantile hemangiomas,^14^ and has been designated an orphan drug for the treatment of glioma and angiosarcoma.^15^ Propranolol is a well‐established drug with a good safety profile and few contraindications.^16^

To the best of our knowledge, a comprehensive evaluation on the effects of propranolol in primary and metastatic UM has not been performed. Here, we confirm the effects of propranolol in CM and demonstrate the first evidence of anti‐tumour effects in UM cells in vitro. Furthermore, a correlation between β1 and β2‐AR expression and aggressiveness in UM tumors from enucleated eyes of patients is shown for the first time.

## 2 METHODS

### 2.1 Cell cultures

Primary human UM cell line MEL270 and metastasis human UM cell line OMM2.5, stem from the same patient and were kindly gifted by Dr Vanessa Morales (University of Tennessee). MP41 and MP46 UM cell lines were acquired from American Type Culture Collection (ATCC, Manassas, VA, USA). WM115 and WM266.4 CM cell lines were derived from primary and metastatic CM, respectively, from the same patient (ATCC). All cultures were maintained in Roswell Park Memorial Institute media (RPMI) + Glutamax (Gibco), 10U/ml penicillin and 10 μg/mL streptomycin (Gibco), except MP46 cell line which was grown in RPMI 1640 high glucose (ATCC) containing 20% of fetal bovine serum (FBS; Gibco). Cultures were incubated at 37°C supplemented with 5% CO_2_ in a humidified incubator. Cells were quantified using a TC20 Automated cell counter (Bio‐Rad). Cells were authenticated before use by IDEXX Bioresearch (USA).

### 2.2 Propranolol treatment

Propranolol hydrochloride (Cat P0884; Sigma‐Aldrich) was dissolved in Dimethyl sulfoxide (DMSO) (Tocris, Oakville, ON, Canada) in a stock solution of 50mM. The solution was diluted freshly prior to each experiment in serum free (SF) RPMI media to different concentrations as indicated in each essay. The concentration 0 stands for SF media with the amount of dissolvent (DMSO) using in the highest concentration. Cell cultures were starved 6 hours (h) prior to treatment using SF RPMI media (Gibco) and maintained at 37°C supplemented with 5% CO_2_ in a humidified incubator.

### 2.3 Morphology

Cells (5 × 10^5^) were seeded in a six‐well plate (Costar). Cell cultures were treated with 0‐200 μmol/L propranolol. Upon 24‐hour propranolol treatment, morphology changes were documented using an EVOS XL microscope (ThermoFisher).

### 2.4 Cell viability assay

Cells (1.5 × 10^4^) containing in 100 μL RPMI media were seeded per well in a 96‐well plate (Costar). Cytotoxicity of propranolol was tested, using doses ranging from 0 to 200 μmol/L. After 24 hours, 10 μL of cell counting kit 8 (CCK8) viability assay (Dojindo Molecular Technologies) was added. The plate was incubated at 37°C and 5% CO_2_ for 1 hour and subsequently read at 450 nm using an infinite M200 Pro plate reader (Tecan Trading AG). Serum free media plus CCK8 solution was used as a blank control. Survival rate was calculated as: absorbance sample − blank/absorbance negative control − blank.

Trypan blue exclusion: Upon propranolol treatment (0, 12.5, 25, 50,100 and 200 μL) for 24 hours. Fifty microliters of cell suspension was mixed with 50 μL of trypan blue stain 0.4% (Gibco). The number of dead and living cells was determined by TC20 Automated cell counter. Experiments were done in triplicate.

### 2.5 Migration assay

Migration was assessed using the scratch wound healing assay. 2 × 10^5^ cells per well were seeded in a six‐well plate until 90%‐100% confluency. Harvested cells were left to adhere for 24 hours with RPMI media supplemented with 10% FBS. Upon cells starvation, a 200 μL pipette tip was used to draw a vertical line down the monolayer of cells across the center of the well. After scratching, the wells were washed twice with PBS1X (Corning cellgro) to remove detached cells and replenished with fresh RPMI media and propranolol (0‐175 µmol/L). Wound induction (0 hour) and cell migration (after 24 hours) were monitored with three images spanning the length of the entire wound using an EVOS XL microscope (ThermoFisher), under phase contrast and magnification of 4×. The measurement of the migration area was analyzed with ImageJ software (National Institute of Health). The migration rate was calculated by: % wound healing = (wound length at 0 h − wound length at 24 h/wound length at 0 h) × 100.

### 2.6 Cell death assay

The In Situ Cell Death Detection Fluorescein (Roche) kit was used to detect cell death after 24‐hour propranolol treatment (0‐200 μmol/L).

3‐5 × 10^4^ cells were transferred with a cytocassette to be spun in a cytocentritifugue (StatSpin Cytofuge 2; Beckman coulter). Manufacturer's instructions were followed. 50 UI of DNase I recombinant (Roche) was used as a positive control. Vectashield Antifade Mounting Medium with DAPI (Vector laboratories) was used to identify nucleus. Images were captured using the Leica DMi8 inverted fluorescence microscope and the Leica LAS X software.

### 2.7 Flow cytometry analysis

Flow cytometry analysis was performed at the Immunophenotyping Platform of the Research Institute of the McGill University Health Centre (MUHC‐RI). Early apoptosis and necrosis were evaluated in cells under propranolol treatment (0, 50, 100, and 200 mmol/L) using Annexin V‐FITC kit (Cell Signaling Technology) following manufacturer's instructions. Unstained cells were used as a negative control. For cell cycle analysis, 8 × 10^5^ to 1.5 × 10^6^ cells were seeded per well in a six‐well plate. Plated cells were then starved during 12 hours in a SF RPMI 1640 media and afterward incubated with 0‐200 µmol/L propranolol. Following the 12‐hour treatment, single cell suspensions were prepared and fixed with ice‐cold 70% ethanol during 1 hour at 4°C. Subsequently, cells were washed with phosphate buffered saline and suspended in 1 µL/mL Ribonuclease A (Thermofisher) for 30 minutes at room temperature. Cells were then transferred into 5 mL polystyrene round‐bottom tubes (Corning), and stained with Propidium iodide (PI) solution (Cat P4170; Sigma) for 30 minutes at 37°C protected from light. Cell cycle was analyzed with BD FACSCanto II system and the PI solution (Sigma) was detected in the blue 488 nm wavelength, 585/42 nm filter.

### 2.8 cell free DNA

cell‐free DNA (cfDNA) in UM and CM cells were detected based on known mutations of each cell lines (Table S1).^17^ 5 × 10^5^ cells per well in a six‐well plate were seeded. Cells were starved 6 hours prior treatment. Upon 24 hours of 0‐200 μmol/L propranolol treatment, cell culture supernatants were collected. cfDNA was extracted from 4 mL‐CM and UM cell culture supernatants using QIAamp Circulating Nucleic Acid kit following urine protocol. The presence of wild type sequence and hotspot mutations (GNAQ (Q209P), GNA11 [Q209L] in UM and BRAF [V600D]) in CM were analyzed in 2 ng of cfDNA quantify by Nanodrop (ThermoFisher). Custom probes and primers were designed by IDT‐Technologies (Integrated DNA Technologies). More detailed PCR conditions in Data S1.

### 2.9 VEGF levels

Vascular endothelial growth factor secretion was evaluated in cell cultures supernatant obtained from primary and metastatic UM and CM cells using a human VEGF Quantikine ELISA kit (R&D System). Cultures were maintained in hypoxic chamber (1% O_2_). Following 24 hours of propranolol treatment (0‐200 μmol/L), supernatants were collected. VEGF levels were determined according to manufacturer's instructions.

#### 2.9.1 Immunostaining

An automated immunohistochemistry was performed in 5 μm sections obtained from UM and CM cell blocks as well as 36 formalin‐fixed paraffin embedded UM cases using 1:100 dilution for β1 and β2‐AR antibodies (Abbiotec). Staining was done on a Ventana autostainer (Benchmark LT). For tumor specimens of UM cases, positivity was evaluated manually by an ocular pathologist (MNB) based on a scale from 0 to 2 (0 = negative, 1 = mild, 2 = intense). Staining extent was classified as 1 = focal or 2 = diffuse. Extent and intensity were converted to the German Immunoreactive Score by multiplying both scores.^18^

##### Analysis of data

All experiments were done in at least three biological replicates with at least four technical replicates for each cell line. Statistical analysis was performed using GraphPad Prism 5 software. Analysis of variance with a post hoc test was performed to compare cell viability, migration, flow cytometry assay as well as evaluate the number of copies of cfDNA and VEGF levels. A value of *P* < .05 was considered statistically significant.

## 3 RESULTS

### 3.1 β‐AR are expressed by UM and CM cells in vitro

**Figure 1:**
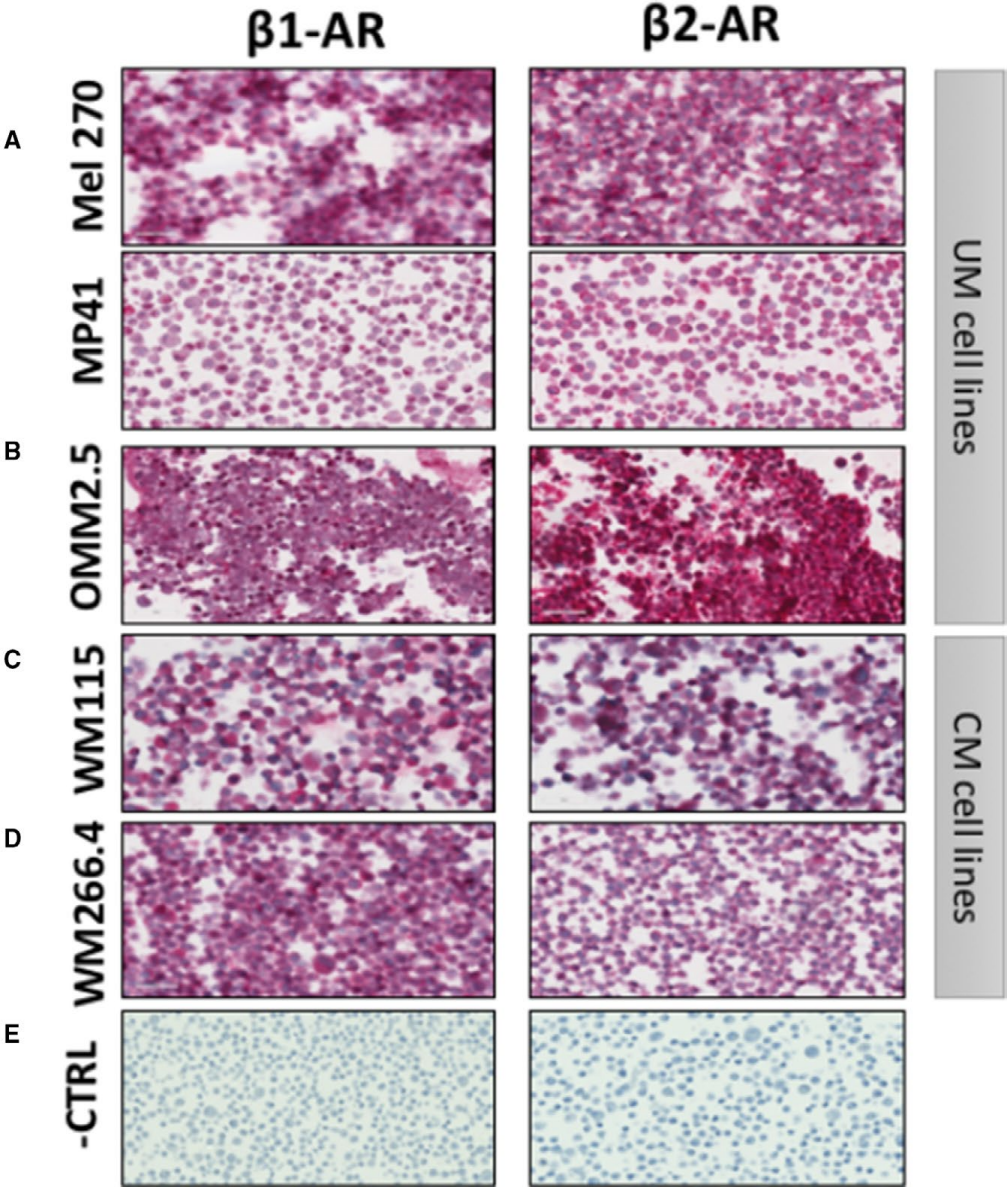
Expression of β‐adrenoceptor (AR) types 1 and 2. Immunohistochemical expression in (A) primary uveal melanoma (UM) cell lines (MEL270 and MP41), (B) metastatic UM cell line (OMM2.5), (C) primary cutaneous melanoma (CM) cell line (WM115) and (D) metastatic CM cell line (WM266.4) (E) Slides stained without primary antibody. Images captured at 40×. Scale = 100 μm

To assess whether β‐blockers could act on melanoma cells, we first sought to determine whether UM and CM cells express β1 and β2‐AR. Immunocytochemistry confirmed the cytoplasmic and nuclear expression of β1 and β2‐AR in cytospins of primary (MEL270 and MP41) and metastatic (OMM2.5) UM cells (Figure 1A,B) and in primary (WM115) and metastatic (WM266.4) CM cells lines (Figure 1C,D). Confocal microscopy confirms β1 and β2‐AR in UM cells (Figure S4A). In contrast, we also evaluated β1 and β2‐AR expression in choroidal melanocytes and primary epidermal keratinocytes, which showed reduced β1 and β2‐AR staining compared to UM cells.

### 3.2 Propranolol affects cellular morphology, proliferation, and migration of melanoma cells in a dose‐dependent manner

**Figure 2:**
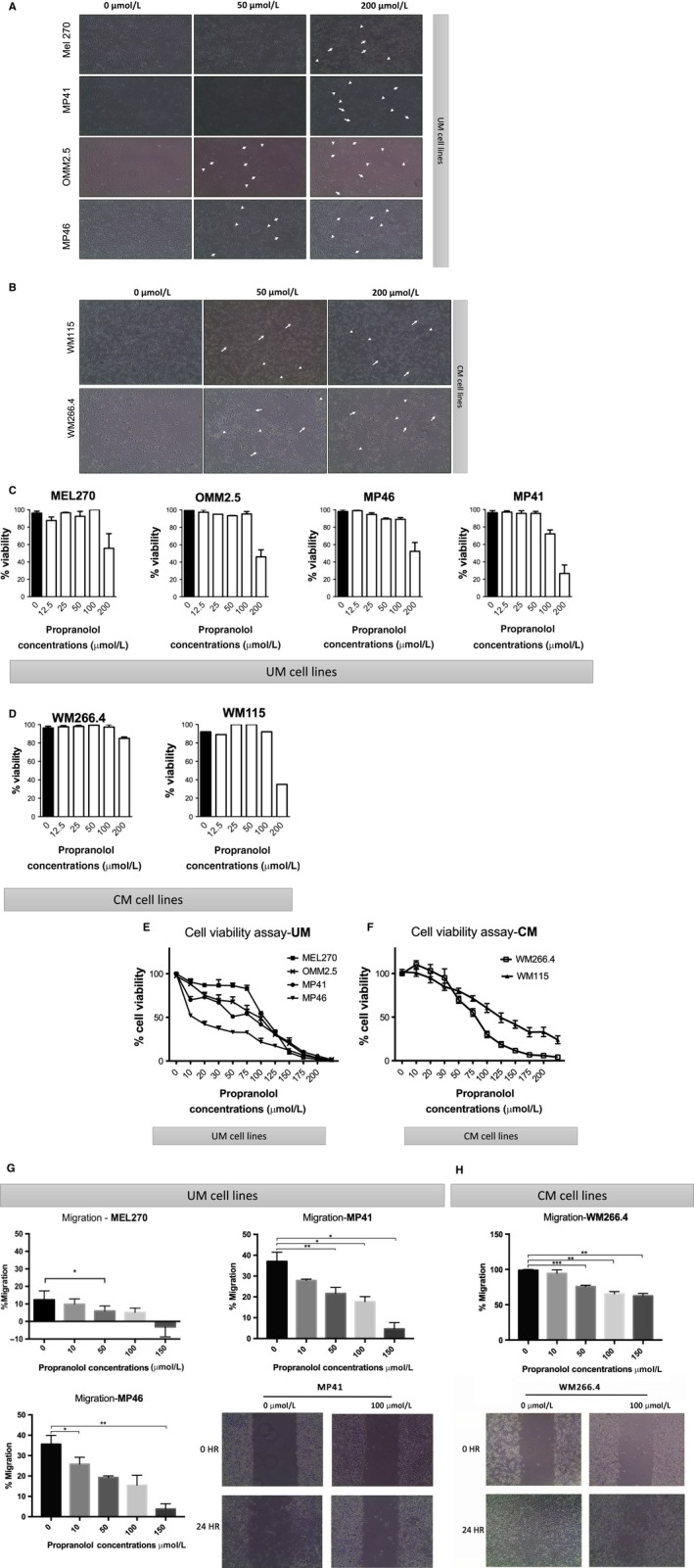
Effect of propranolol in uveal melanoma (UM) and cutaneous melanoma (CM) primary and metastatic cell lines. Representative images of cellular shape changes under 50 and 200 μmol/L propranolol treatment in (A) UM cell lines and (B) CM lines. 20× objective. Viability is shown by tryptan blue exclusion assay in triplicates in UM (C) and CM (D) cells. Cytotoxicity of propranolol at different concentrations in (E) UM and (F) CM cell lines is shown using CCK8 viability assay. Relative cell viability % was calculated as compared with the 0 μmol/L (no drug) control (100% viability). Error bar shows ± SD. Dose‐dependent inhibitory effects of propranolol on the percentage of migration of (G) UM (MP41, MP46, MEL270) and (H) CM (WM266.4 and WM115) cell lines were detected by wound healing assay after 24 h of drug exposure. *P < .05 vs control (0 μmol/L). Representative images of UM (MP41) and CM (WM266.4) migration assays (under phase contrast, 4× objective) are shown

The effects of propranolol treatment on UM and CM cells in vitro were assessed. Morphology alterations were documented upon 24 hour treatment in cultured UM cell lines (Figure 2A) and CM lines (Figure 2B). Under propranolol exposure, all UM and CM cells studied underwent two morphological changes: (a) stretching of the cytoplasm, where cells remain adhered to the cell culture plate; and (b) cells become rounded and loose, in particular under 100 µmol/L or higher dose. While the number of cells did not change during the 24 hour propranolol treatment compared to control‐treated cells (Figure S1), we observed that cell viability by trypan blue staining decreased with propranolol treatment in both UM (Figure 2C) and CM (Figure 2D) cells at 24 hours. This was confirmed by CCK8 in a dose‐dependent manner of propranolol treatment in UM cells (Figure 2E) and CM (Figure 2F) cell lines after 24 hours. A reduction in dehydrogenase activity by 50% occurred with 100 μmol/L propranolol in UM and CM cells, suggesting a reduction in metabolic activity. Similarly, cell migration was reduced in both UM (Figure 2G) and CM (Figure 2H) cells following drug treatment, and this was related to drug dose. Interestingly, we also tested cell viability by CCK8 in normal choroidal melanocytes and keratinocytes (Figure S3A,B). Our findings indicate less toxicity to normal cells than UM and CM cells. In choroidal melanocytes that we cultured from human donor eyes, propranolol up to 50 μmol/L does not decrease cell survival, while 100 μmol/L decreases cell viability around 30%, and 200 μmol/L has a cytotoxic effect. In keratinocytes, no tested dose had an impact on viability.

### 3.3 Propranolol has a cytotoxic effect via induction of apoptosis and cell cycle arrest

**Figure 3:**
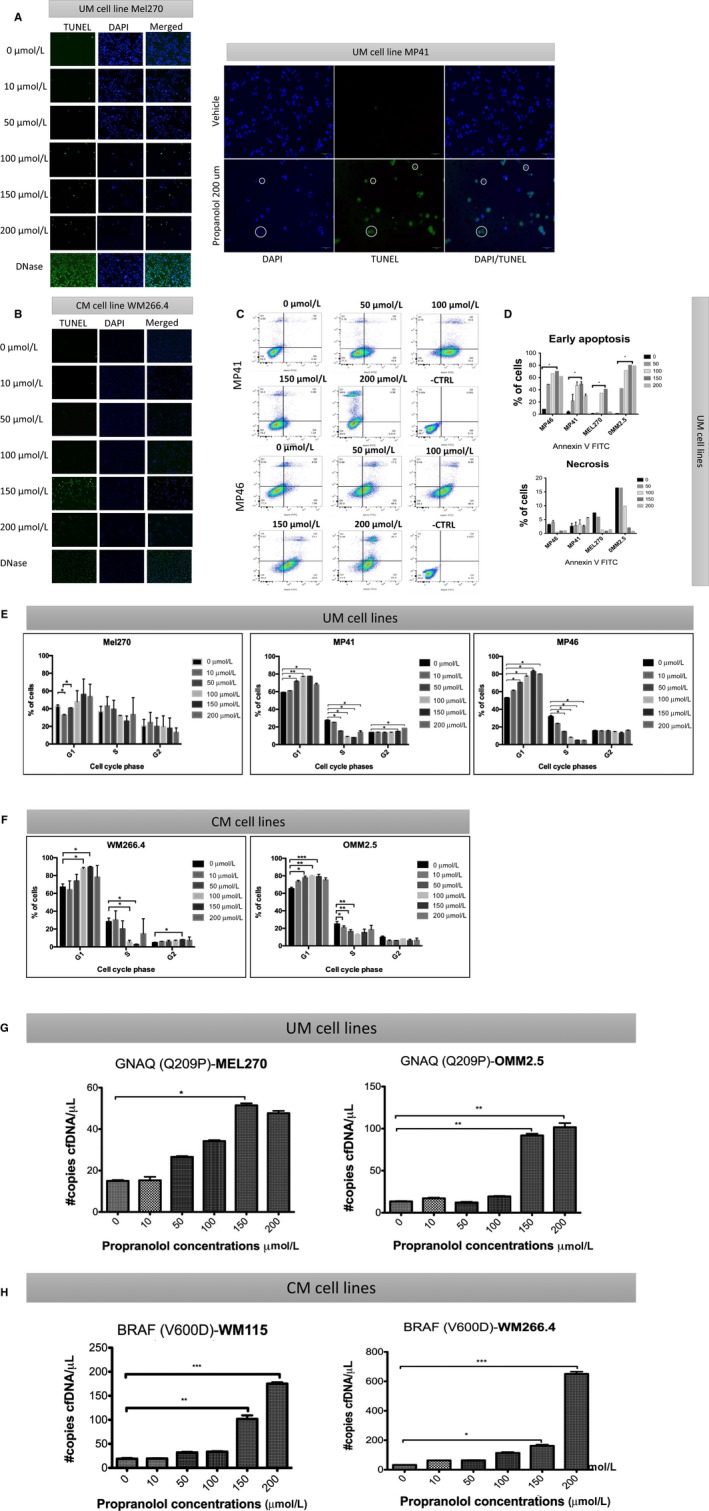
The effect of propranolol on apoptotic cell death and cell cycle arrest in melanoma cells. TUNEL staining showed the presence of apoptotic cells under 50, 100, 150, and 200 μmol/L propranolol exposure during 24 h in (A) uveal melanoma (UM) (Mel 270 and MP41) and (B) cutaneous melanoma (CM) (WM266.4) cells. Cells under DNase were used as a positive control. Green color indicates TUNEL‐positivity, blue color marks nucleus. 20× objective. Representative flow cytometry analysis of UM using Annexin V FITC (C). Bar graph of early apoptosis, necrosis, and cell death on cultured UM cells (D). Cell cycle analysis was done by flow cytometry following propranolol exposure at 6 concentrations in UM (E) and CM (F) cell lines. Error bars represent ± 1 SD. *P < .05 vs control (0 μmol/L). cell‐free DNA (cfDNA) in the supernatant was assessed following 24 h treatment with propranolol in (G) UM (Mel 270 and OMM2.5) and (H) CM (WM266.4 and WM115) cell lines. The specific mutation for each cell line is indicated. *P < .05 vs control (0 μmol/L)

Previous studies have shown the pro‐apoptotic role of propranolol in CM cells and in vivo murine model.^19^ To assess whether propranolol induces apoptotic cell death in UM and confirm this in CM, a Terminal deoxynucleotidyl transferase dUTP nick end labeling (TUNEL) assay was performed following propranolol treatment. As shown in Figure 3, TUNEL‐positive cells (green) were detected at 24 hours under >50 μmol/L propranolol treatment on UM (Figure 3A) and CM (Figure 3B) cell lines. To assess cell death at a cell population level, an Annexin V FITC assay was performed. Cell populations of Annexin V FITC positive and PI negative expression were detected in a dose dependent manner. Likewise, some cells were Annexin V FITC negative and PI positive which indicates necrosis under 24 hours propranolol treatment (Figure 3C,D). To confirm that cells were undergoing apoptotic cell death, DNA strands were identified by labeling of 3′OH termini with modified nucleotides and catalyzed by the action of terminal deoxynucleotidyl transferase in MP46 cells as an example (Figure S2A). Chromatin staining with DAPI revealed that vehicle‐treated MP46 nuclei displayed evidence of chromosome arrangement suggesting cells transitioning into mitosis, while most of the cells showed relaxed chromatin (Figure S2B). In contrast, during propranolol treatment (100 μmol/L), cell nuclei displayed more condensed chromatin with no evidence of chromosome arrangement, suggesting that cells were arrested in the cell cycle. Apoptotic bodies are also observed. To rule out that propranolol was inducting senescence in UM and CM cell lines, we performed a β‐Galactosidase staining assay after 24 hour propranolol treatment (0‐200 μmol/L). The results showed no induction of senescence in the UM and CM cell lines tested (Figure S5).

Propranolol has been shown to induce G0/G1/S phase arrest and apoptosis in melanoma cells via the AKT/MAPK pathway.^19^ To determine the effects of propranolol on UM and CM cells in our model, we performed flow cytometric analysis of cell cycle following drug exposure. UM (Figure 3E) and CM (Figure 3F) cells showed arrest at G1, with decreasing proportions of cells in S phase.

### 3.4 cfDNA as an indicator of treatment response

To assess whether increased cell death due to propranolol would result in increased secretion of cfDNA into the cell culture media supernatant, we quantified cfDNA from cell lines after treatment using ddPCR for mutations in GNAQ (Q209P) and GNA11 (Q209L) in UM cells and BRAF (V600D) in CM cells. The release of cfDNA quantified by the number of copies per reaction correlated with propranolol concentration, with increasing cfDNA levels following higher propranolol dose exposure 24 hours post‐treatment in UM (Figure 3G) and CM (Figure 3H) cells.

### 3.5 Propranolol decreases VEGF secretion in primary human UM cells

**Figure 4:**
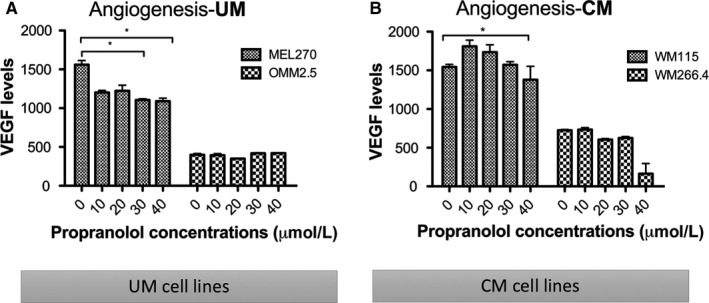
Effect on vascular endothelial growth factor (VEGF) production after propranolol treatment. VEGF production was assessed by ELISA after 30 and 40 μmol/L of propranolol in (A) primary uveal melanoma (UM) (MEL270) and (B) primary cutaneous melanoma (CM) (WM115) cells after 24 h treatment. *P < .05 vs control (0 μmol/L)

Previous studies have shown that β‐blockers exert primary downstream effects that include vasodilation and release of pro‐angiogenic factors, such as VEGF.^5^ As such, we aimed to assess the effect of propranolol on VEGF production in vitro. Vascular endothelial growth factor levels in the cell culture supernatant of UM (Figure 4A) and CM (Figure 4B) cells were significantly reduced after 24 hour‐treatment with propranolol (40 μmol/L) compared to no treatment (*P* < .05).

### 3.6 Expression of β‐AR in UM patient samples

**Figure 5:**
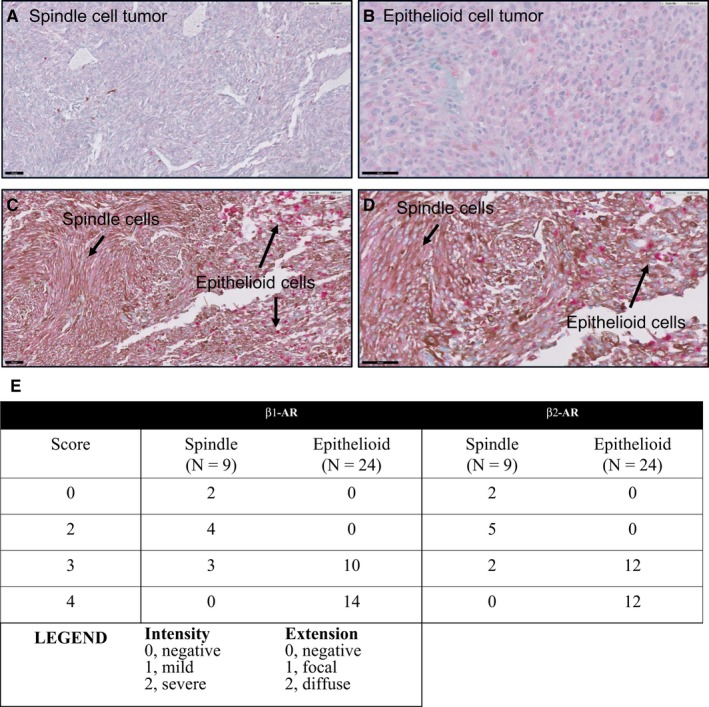
Expression of β‐adrenoceptors in clinical UM samples. A, Spindle cell tumor showing focal areas of immunostaining (intensity 1, extension 1 = grade 2). B, Epithelioid cell tumor showing diffuse and intense staining (intensity 2, extension 2 = grade 4). (C, D) Same tumor with areas of spindle cell staining and epithelioid cell staining. Immunostaining was done using a red chromophore to differentiate from melanin pigment (brown). Scale 50 μm

Finally, β‐AR expression has been shown in tumor tissue of CM patients.^10^ To determine whether clinical samples of UM displayed expression of these receptors, we performed immunohistochemical analysis of β1 and β2‐AR in 36 enucleated UM cases. Of these cases, 3 were excluded (one necrotic tumor, two prior irradiated tumors). Of the 33 remaining cases, nine were classified as spindle cell, seven as epithelioid cell, and 17 as mixed cell type; however, all 17 mixed showed >70% epithelioid cells and were therefore classified as epithelioid (n = 24 total). Beta‐adrenoceptors expression was evaluated using an multiplying score combining intensity and extension of positivity, and summarized in Figure 5E. Epithelial cell tumors showed higher expression of β1 and β2‐AR than spindle cell tumors (*P* < .001; Figure 5A,B,E). Within a mixed cell type tumor, areas of spindle cell staining (grade 2) and epithelioid cell staining (grade 4) can be seen (Figure 5C,D). This is the first analysis of the expression of β‐AR in UM patient tissue.

## 4 DISCUSSION

To the best of our knowledge, this is the first analysis of the effects of β‐blockers in UM cells. Our results show consistent anti‐proliferation, reduced migration, and pro‐apoptotic effects of propranolol in several UM and CM cell lines. We also report the first analysis of the expression of β1 and β2‐AR in tumors from UM patients and in human UM cell lines. Collectively our data suggest that further pre‐clinical studies are warranted to assess whether the use of beta‐blockers may be effective to treat UM.

The results of the present study are supported by reported data on the effects of β‐blockers on cancer cell lines. Previous work has shown that beta‐blockers can inhibit the pro‐tumor effects of β1 and β2‐AR, such as proliferation, migration, angiogenesis, resistance to anoikis, as well as downregulate the expression of pro‐tumor molecules such as angiogenic factors and interleukins.^6^, ^20^, ^21^, ^22^, ^23^, ^24^, ^25^

Several groups have previously attempted to elucidate the mechanisms underlying propranolol's anti‐proliferative and pro‐apoptotic effects. β‐AR 1, 2, and 3 can activate ERK/MAPK1/3.^26^ Propranolol induces apoptosis in CM cell lines^19^ through non‐selective β1 and β2‐AR antagonism that may inhibit ERK/MAPK1/3 as well as negatively block the ERK/MAPK pathway by releasing intracellular calcium.^27^ Increased intracellular Ca^2+^ in CM can induce the WNT signaling cascade, responsible for activating PKC and CamKII.^28^ In UM, among the downstream effectors of GNAQ/11, the main function of the second messenger IP3 is to activate PKC by increasing the cytosol calcium levels.^29^ The activation of PKC stimulates the mitogenic RAF/MEK/ERK pathway, an essential driver of proliferation, migration and survival in both CM and UM.^26^, ^30^ This evidence suggests a possible link to explain why propranolol could be beneficial in both UM and CM. Interestingly, previous studies have shown that propranolol lacks toxicity to normal cutaneous melanocytes.^31^  We also demonstrated similar safety of propranolol to choroidal cells.

Preclinical studies have also shown that β‐blockers can inhibit migration and angiogenesis of cancer cells through inhibition of noradrenaline‐dependent pathways.^32^ Using an ELISA assay, we observed a decrease in VEGF levels in primary UM and CM cell lines after propranolol exposure but no difference was observed in metastatic cell lines. Norepinephrine has been found to increase angiogenic factors,^25^ while propranolol has been shown to decrease VEGF levels.^33^ Assessment of other angiogenic markers in UM and CM cells treated with propranolol could further elucidate the angiogenic response to β‐blocker treatment.

This is the first study that uses cfDNA as an indicator of propranolol treatment response. cfDNA release into supernatant has been documented in cancer cell cultures.^34^ We observed that cfDNA concentration increased in a dose‐dependent manner upon 24‐hour propranolol treatment. Interestingly, the amount of cfDNA correlated with the number of cells in G1 cell cycle arrest, which coincides with a previous report.^35^ Further analysis in the origin of the releases of cfDNA in vitro is needed to understand its mechanism.

Clinical studies have shown promising data on the anti‐tumoral effect of propranolol,^36^, ^37^, ^38^ including for the treatment of melanoma. Patients on β‐blockers had lower melanoma progression as compared to patients not on β‐blockers,^39^ suggesting a therapeutic benefit in this tumor setting. A prospective clinical study showed that propranolol was inversely associated with recurrence.^40^

Current evidence suggests the combination of drugs capable of regulating multiple pathways may improve disease control.^7^ Therefore, it is crucial to develop new sustainable and effective adjuvant therapeutic options. Drug repurposing studies are a cost‐effective means to find new applications to approved drugs with good safety profiles.

Data on the anti‐tumor effects of propranolol by our group and others therefore provide a promising avenue for effective combination or alternative treatments for melanoma patients. Propranolol's proven safety record and cost‐effectiveness, as well as its anti‐tumor effects and immunotherapy potentiating attributes^41^ could provide an attractive mechanism to treat a wide range of patients with melanoma. Our results demonstrate a clear effect of propranolol on melanoma cells, further supporting the growing body of literature on the effects of β‐blockers on CM, and for the first time showing potent effects in UM. Propranolol inhibits proliferation and migration, decreases VEGF production and induces melanoma cell death and cell arrest in G1 phase. Furthermore, cfDNA seems to be a treatment‐response biomarker that could be used in other treatments. This study shows for the first time that β1 and β2‐AR are expressed in human UM. Studies into the clinical benefit of propranolol in UM could help to develop a novel adjuvant therapy for this deadly disease.

## 5 SIGNIFICANT CONCLUSIONS

For the first time, we show potent anti‐tumor effects in UM cells following propranolol administration and positive expression of β1 and β2‐AR in all human UM specimens, correlating with aggressiveness of the tumor. Collectively our data suggest that a non‐selective β‐blocker may be effective against melanoma, and further studies are warranted to validate this as an adjuvant therapy in melanoma.

## CONFLICT OF INTEREST

None.

## AUTHOR CONTRIBUTIONS

PB and DM designed and conducted experiments, analyzed data, and helped draft the manuscript. PGAG, EJ, TT, AG, and ABD conducted experiments and reviewed the manuscript. MNB scored the pathology and reviewed the manuscript. JVB oversaw all experiments and analyses and wrote the manuscript.
