# Supplementary figures and images for: Beta‐blockers exert potent anti‐tumor effects in cutaneous and uveal melanoma
Source: Cancer Med. 2019 Oct 7;8(17):7265–77. doi: 10.1002/cam4.2594 (PMC6885887; doi:10.1002/cam4.2594)

Supplementary Figure 1A

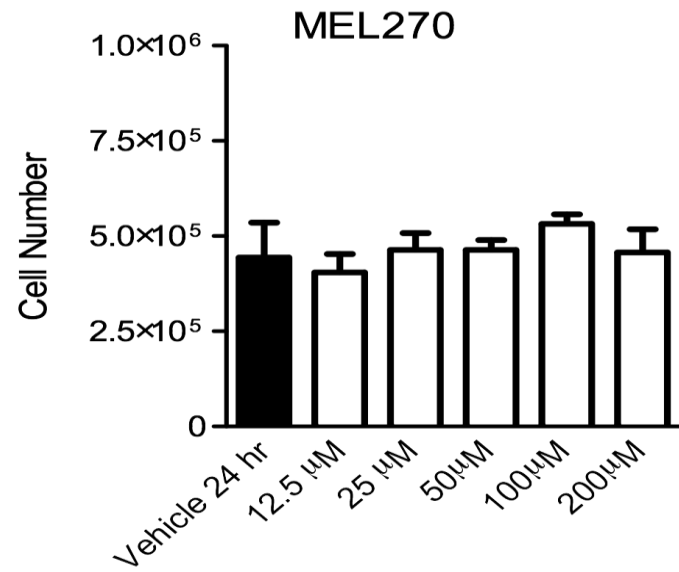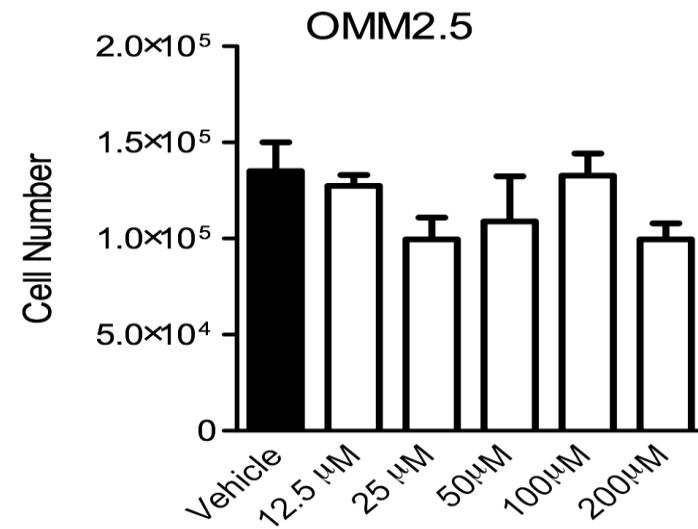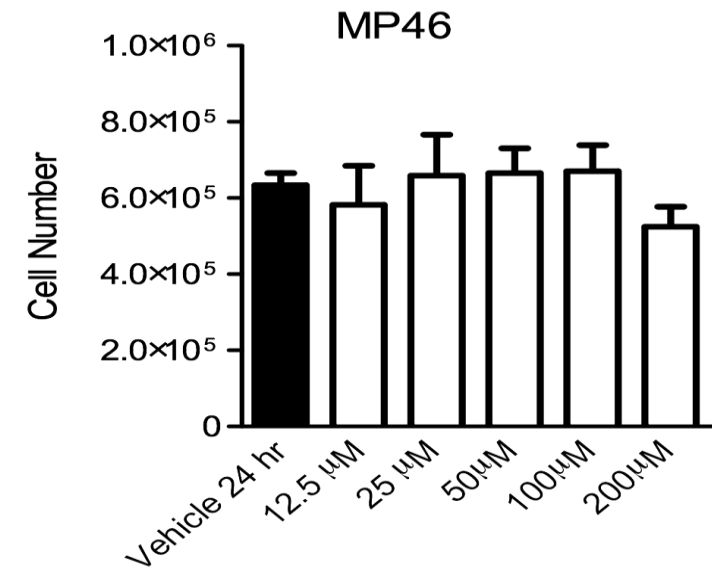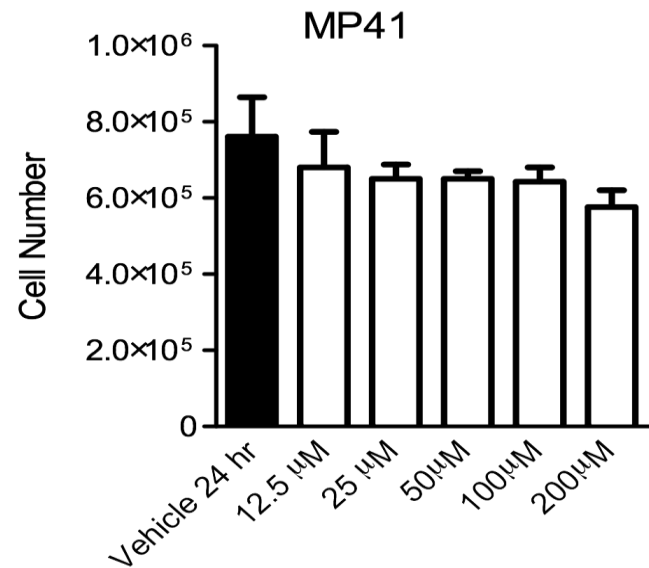

Supplementary Figure 1B

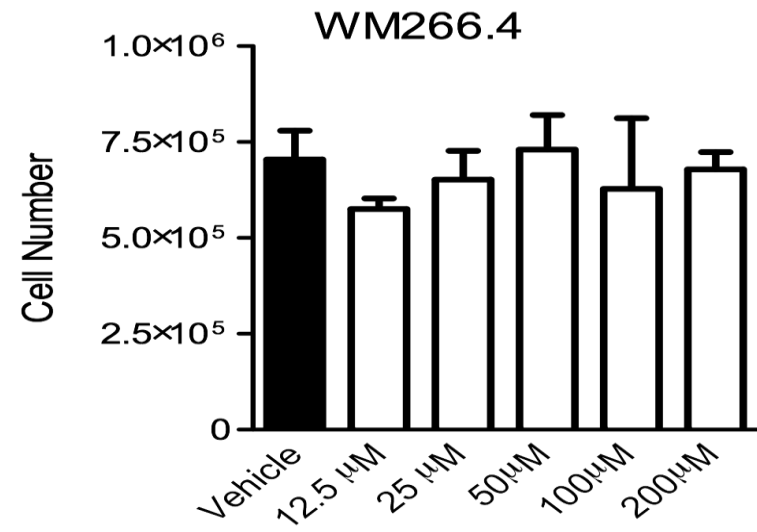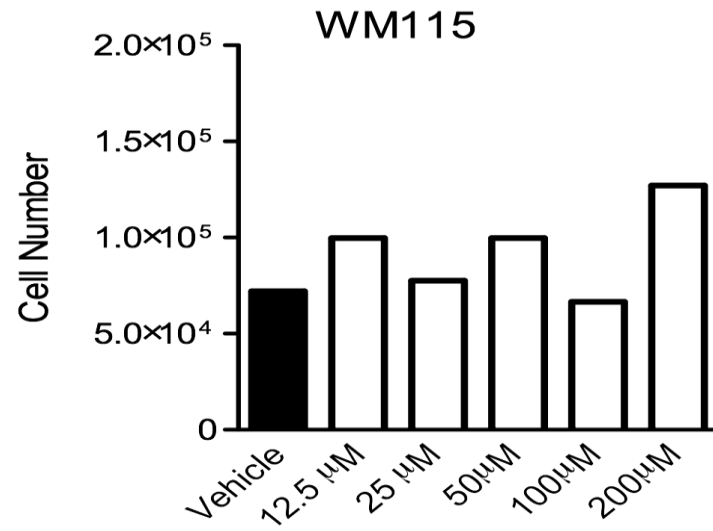

Supplement: Supplementary file 1 [file CAM4-8-7265-s001.pdf]

## Supplementary Figure 2A

UM cell line MP46

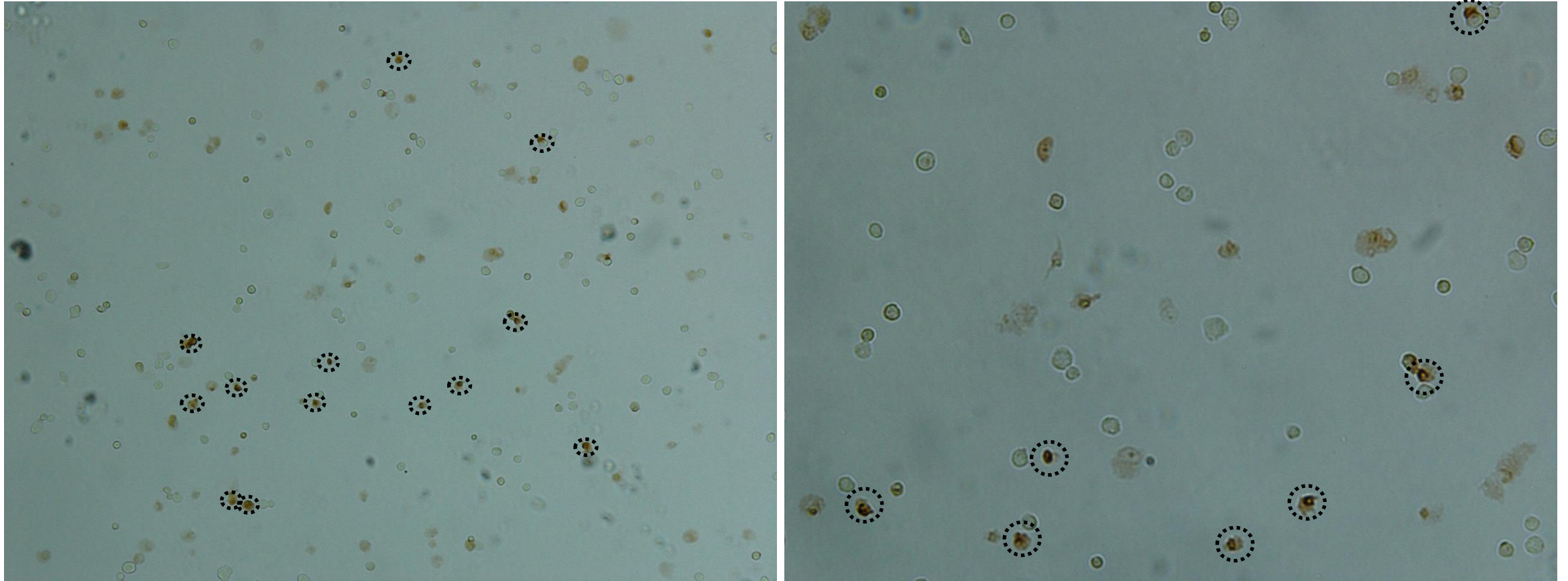

**Propranolol: 200 $\mu$ M**

Supplementary Figure 2B

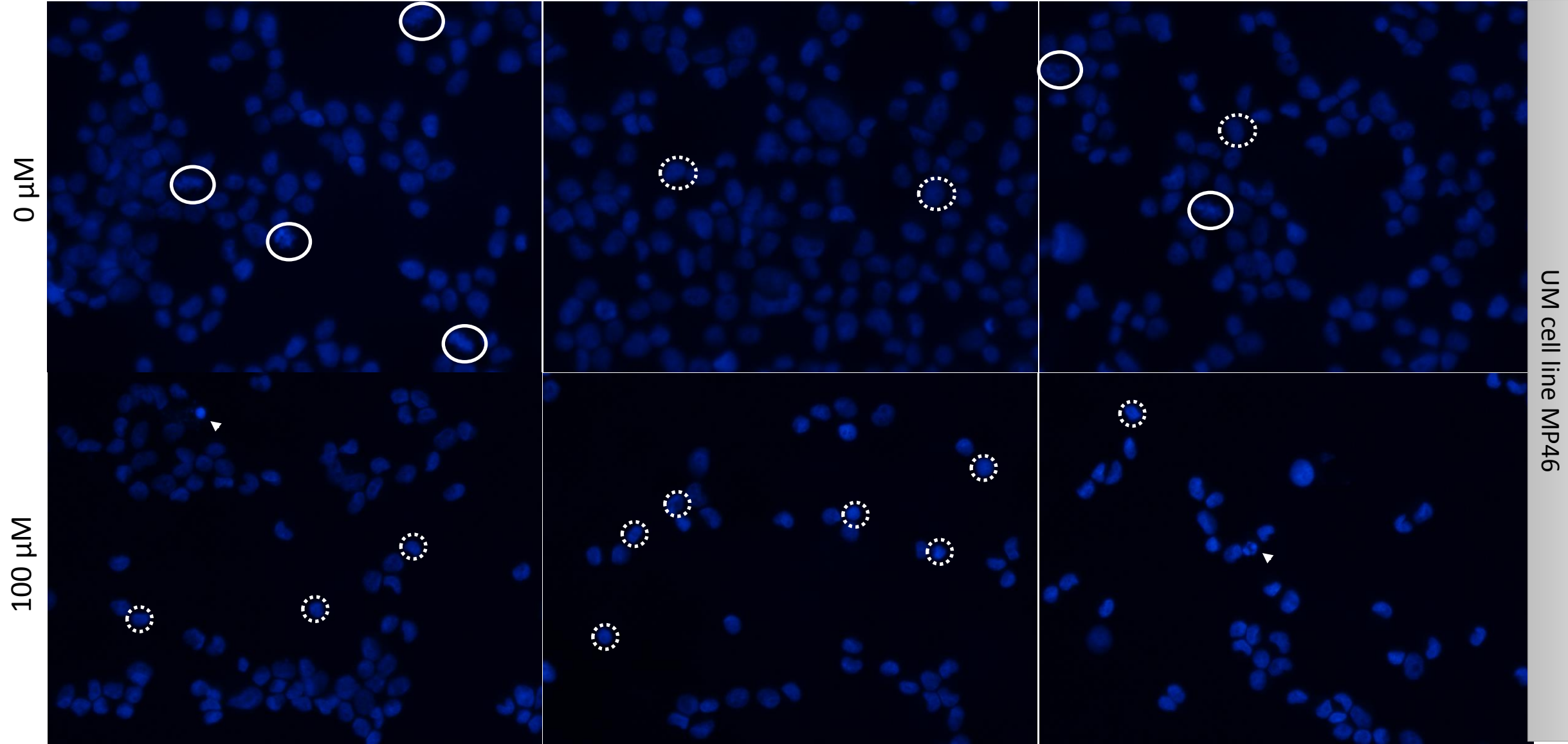

Supplement: Supplementary file 2 [file CAM4-8-7265-s002.pdf]

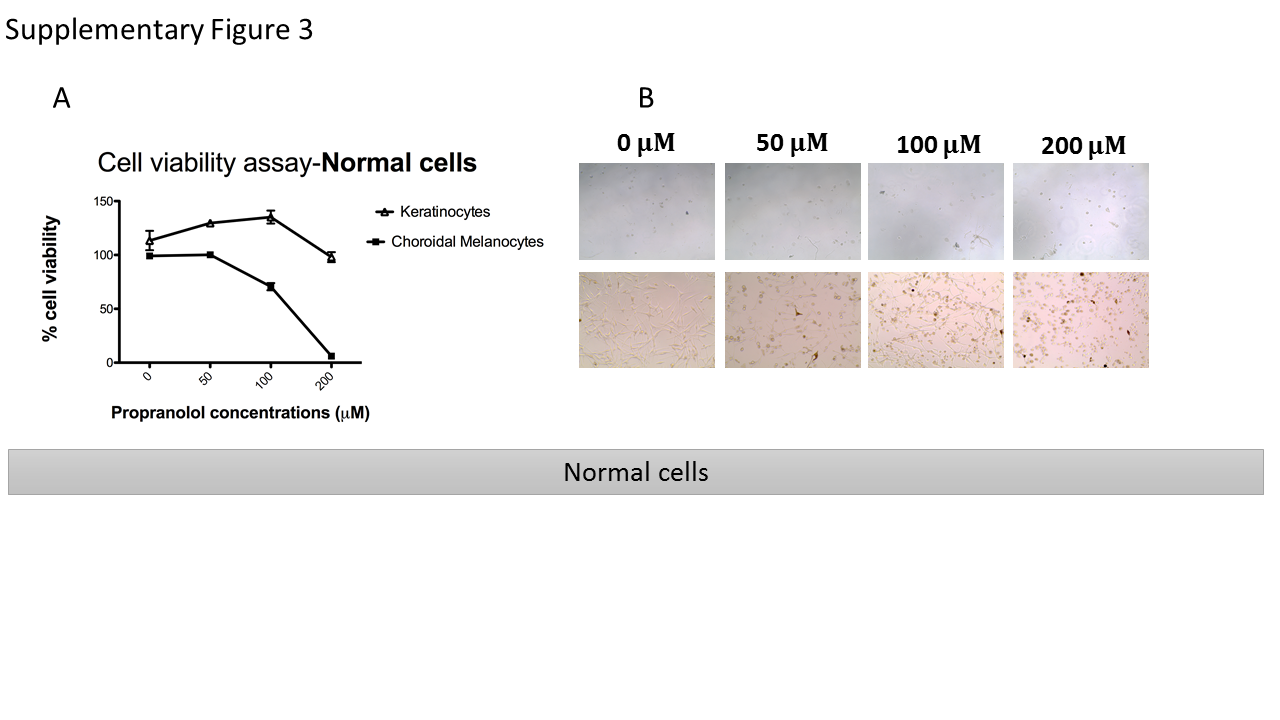

Supplement: Supplementary file 3 [file CAM4-8-7265-s003.tif]

Supplementary Figure 4A

$\beta 1$  and  $\beta 2$  AR expression

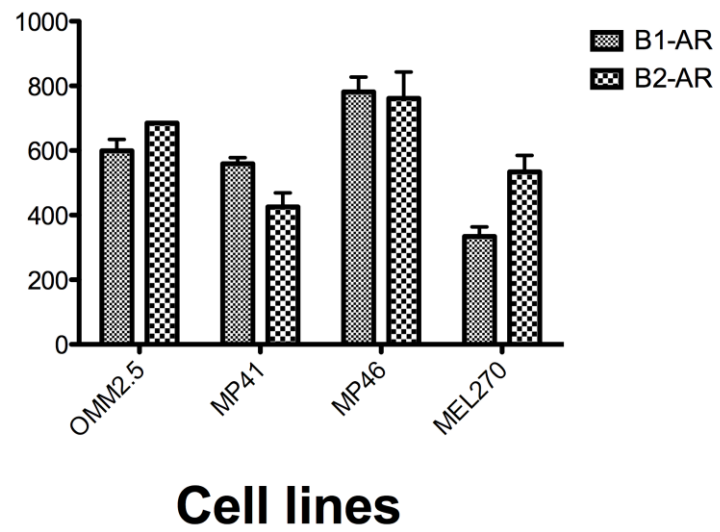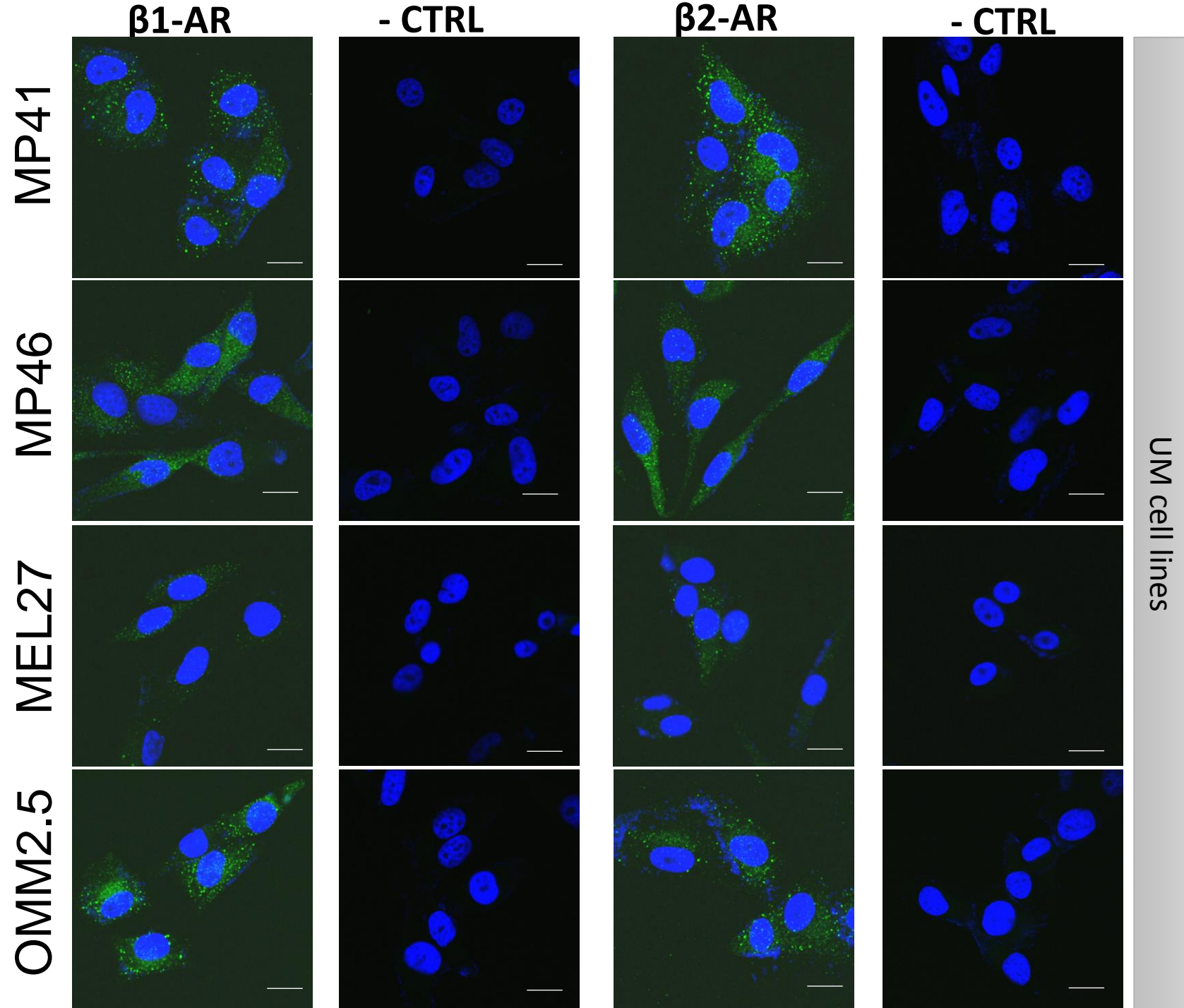

Supplementary Figure 4B

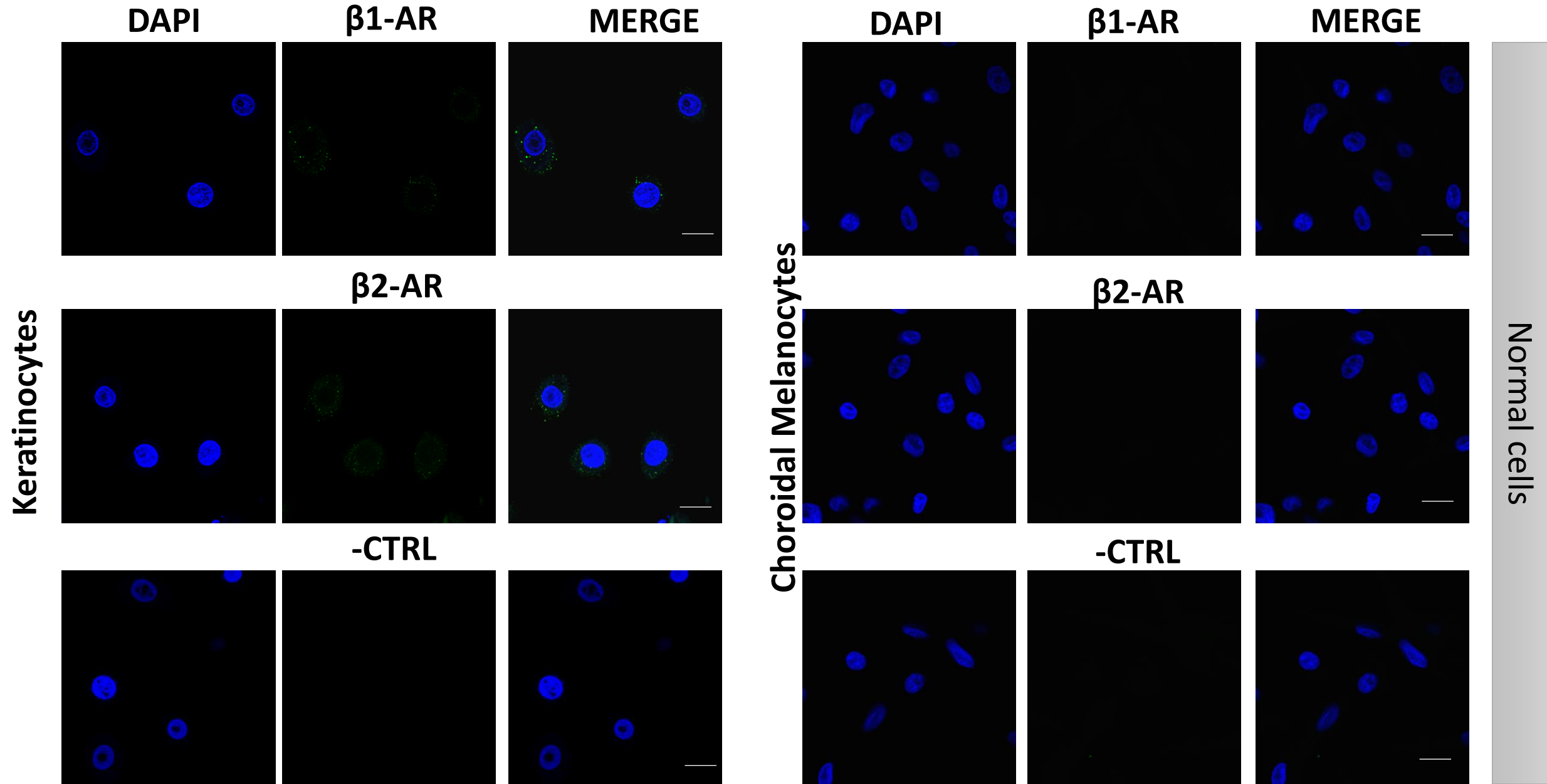

Supplement: Supplementary file 4 [file CAM4-8-7265-s004.pdf]

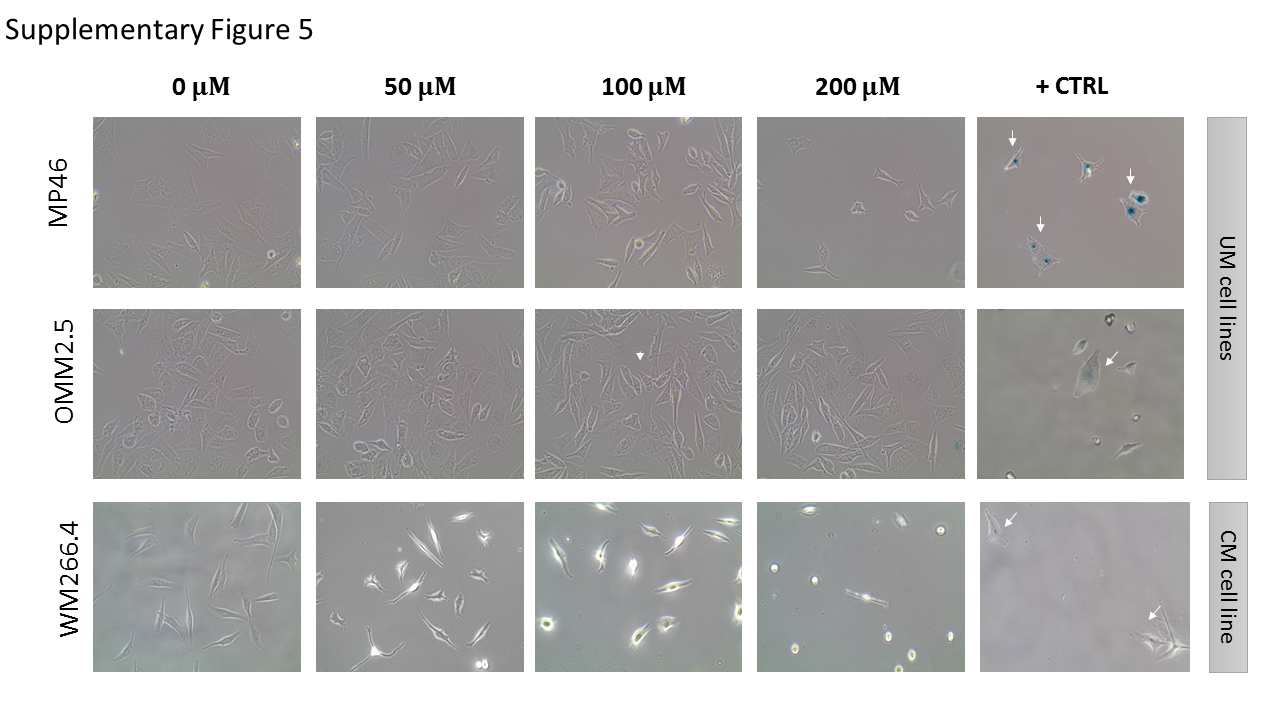

Supplement: Supplementary file 5 [file CAM4-8-7265-s005.tif]
