# Supplementary material for: Beta‐blockers exert potent anti‐tumor effects in cutaneous and uveal melanoma
Source: Cancer Med. 2019 Oct 7;8(17):7265–77. doi: 10.1002/cam4.2594 (PMC6885887; doi:10.1002/cam4.2594)
Supplement: Supplementary file 6 [file CAM4-8-7265-s006.docx]

**Supplementary material**

**Supplementary Methods**

Normal cell lines: Normal Epidermal keratinocytes were purchased from acquired from American Type Culture Collection (ATCC). Under the approval of the ethic committee (RB#2019-5314) Choroidal melanocytes were isolated from a pair of donor eyeballs following two protocols previously described (*Weidmann C et al. S Mol Vis. 2017 12;23:103-115. Differential responses of choroidal melanocytes and uveal melanoma cells to low oxygen conditions* & Valtink and Elgelmann [Graefes Arch Clin Exp Ophthalmol.](https://www.ncbi.nlm.nih.gov/pubmed/17458555) 2007 Oct;245(10):1487-94. Epub 2007 Apr 26. Serum-free cultivation of adult normal human choroidal melanocytes). Choroidal melanocytes were maintained in Melanocyte media M2 (PromoCell, Germany), growth media M2 suplementalMix (PromoCell) and Geneticin (G418, Gibco) at standard conditions.

PCR: PCR reactions containing: 450 nM probes, 900 nM primers, 1X supermix for probes no dUTP (Bio-Rad, Irvine, CA, USA) and samples were performed in a C100 Touch Thermal Cycler (Bio-Rad) using ddPCR 96-well plates (Cat 12001925, Bio-Rad) under the cycling conditions: 95ºC for 10 min, followed by 50 cycles: 95ºC for 30 seconds, 60ºC (GNAQ/11) or 54ºC (BRAF V600D) for 1 minute, 72ºC 30 seconds, finally 98ºC for 10 minutes. Then, plates were analyzed in a QX200 PCR platform (Bio-Rad, Irvine, CA, USA) for ddPCR. 5’Hexachloro-fluorescein (HEX) was used for wildtype (wt) allele and 5’6- fluorescein amidite (FAM) for mutant allele (mut). Number of copies per μl were obtained using QuantaSoft software (Bio-Rad).

Senescence Assay: Upon 24 h propranolol treatment (0, 50, 100 and 200 μM) senescence was evaluated using Senescence β-Galactosidase Staining Kit (Cell Signaling Technology, UK) following kit’s instructions. Senescence induced by stress using hydrogen peroxide was used as a positive control (NN Hooten and MK Evans, J Vis Exp. 2017; (123): 55533).

**Immunofluorescence**

Cells were cultured on chambered cell culture slides (Falcon, Corning, NY, USA) fixed with 4% paraformaldehyde for 30 min. Cells were washed with PBS 1X and blocked in 10% BSA (Bovine serum albumin, Cedarlane, ON, Canada), 10% donkey normal serum (abcam) and 0.1% tween (American Chemicals, NCm USA). Cells were stained with 1:100 dilution for β1 and β2-AR antibodies (Abbiotec, USA) overnight at 4ºC. After washing with PBS 1X, 1:500 Alexa 488 anti-rabbit antibody (Invitrogen, Carlsbad, USA) was incubated for 2h. Slides were mounted with Vectashield Antifade Mounting Medium with DAPI (Vector) and viewed in a Zeiss LSM780 confocal microscope. Cells without primary antibody was used as a negative control.

**Supplementary Table 1.** Mutational characteristics of UM and CM cells

| Cell line | Cell type | Primary/met | Mutation |
| --- | --- | --- | --- |
| Mel270 | UM | Primary | GNAQ Q209P (626 A>C) |
| OMM2.5 | UM | Metastasis | GNAQ Q209P (626 A>C) |
| MP41 | UM | Primary | GNA11 Q209L (626 A>T) |
| MP46 | UM | Primary | GNAQ Q209L (626 A>T) |
| WM115 | CM | Primary | BRAF V600D (1799_1800 TG>AT) |
| WM266.4 | CM | Metastasis | BRAF V600D (1799_1800 TG>AT) |

**Supplementary Figure 1** Viability is shown for UM **(A)** and CM **(B)** cells by cell counting in TC20. Triplicate experiments are shown. Error bar shows ±1SD.

**Supplementary Figure 2. (A)** DNA strand breaks were identified by labeling 3’-OH termini with modified nucleotides and catalyzed by the action of the terminal deoxynucleotidyl transferase in MP46 cells. Biotinylated nucleotide is incorporated at the 3´-OH DNA ends using the Terminal Deoxynucleotidyl Transferase, Recombinant, (rTdT) enzyme. Horseradish peroxidase-labeled streptavidin (Streptavidin HRP) is then bound to these biotinylated nucleotides, which are detected using the stable chromogen, diaminobenzidine (DAB). Using this procedure, apoptotic nuclei are stained dark brown and visualized with a light microscope.  **(B)** Chromatin staining with DAPI in MP46 cells. Solid lines indicate chromosome arrangement observed in non-treated nuclei. Dotted lines represent the chromatin condensation status of non-treated vs. propranolol treated. Arrowheads indicate apoptotic bodies.

**Supplementary Figure 3. (A)** Cytotoxicity of propranolol (0-200 μM) was tested using CCK8 in normal epithelial keratinocytes and choroidal melanocytes. **(B)** Images of normal cells after 24 h propranolol treatment (0-200 μM).

**Supplementary Figure 4. (A)** Fluorescence staining graph and confocal expression in UM cell lines and **(B)** normal keratinocytes and choroidal melanocytes cells. β1 and β2 adrenoreceptor expression is shown in green and the nucleus in blue (DAPI).

**Supplementary Figure 5.** Senescence β-galactosidase staining of UM cells accompanied with positive controls.
